# Contextualising and challenging under-representation in research in light of Cultural Trauma: a qualitative focus group and interview study

**DOI:** 10.1186/s40900-024-00600-3

**Published:** 2024-07-02

**Authors:** Kate Fryer, Isobel Hutt, Habiba Aminu, Emma Linton, Johanna White, Josie Reynolds, Caroline Mitchell

**Affiliations:** 1 Population Health, Research Associate, University of Sheffield, Sheffield, UK; 2 Medical Student, University of Sheffield, Sheffield, UK; 3 Population Health, Academic Clinical Fellow, University of Sheffield, Sheffield, UK; 4 Deep End CRN, Research Nurse, Sheffield, UK; 5 Professor of Primary Care, Keele University, Keele, UK

**Keywords:** Deprivation, Minority, Diverse, Inclusion, Participation, Representation, Inequality, Inequity, Primary care, Underserved

## Abstract

**Background:**

Although underserved populations— including those from ethnic minority communities and those living in poverty—have worse health and poorer healthcare experiences, most primary care research does not fairly reflect these groups. Patient and public involvement (PPI) is usually embedded within research studies in the United Kingdom (UK), but often fails to represent underserved populations. This study worked with patient and public contributors and local community leaders, situated in a socio-economically deprived and ethnically diverse urban area, to explore under-representation in primary healthcare research.

**Methods:**

We undertook a focus group with a purposive sample of 6 members of a Patient and Public Involvement Group (PPIG), and interviews with 4 community leaders (representing Black, South Asian, Roma and socio-economically deprived communities). An iterative analysis process based on template analysis was used. Focus group 1 was rapidly analysed, and a template created. Findings were presented in focus group 2, and the template further developed. The Cultural Trauma concept was than applied to the template to give a wider theoretical lens. In-depth analysis of focus groups and interviews was then performed based on the template.

**Results:**

Wider societal and historical influences have degraded trust in academic and healthcare institutions within underserved populations. Along with more practical considerations, trust underpins personal motivations to engage with research. Researchers need to invest time and resources in prolonged, mutually beneficial engagement with communities of importance to their research, including sharing power and influence over research priorities. Researcher reflexivity regarding differential power and cultural competencies are crucial. Utilising participatory methodologies including co-production demonstrates a commitment to inclusive study design.

**Conclusions:**

Re-framing evidence-based medicine to be more useful and relevant to underserved populations with the highest burden of ill health is urgently needed. Lack of representation in primary healthcare research reflects wider societal inequalities, to which Cultural Trauma provides a useful lens. However, there are actions that researchers can take to widen representation. This will ultimately help achieve the goal of increased health equity by enhancing scientific rigour and research generalizability.

## Abstract (plain language summary)

**The problem:**

People living in poverty, and people from ethnic minority communities may be referred to as ‘underserved’. Underserved communities benefit less from health services, and along with other factors, this leads to health inequalities. Primary care research does not include enough people from these communities. This makes the health inequalities  worse.

**What we did:**

This study looks at why people from underserved communities are not included in research. It also looks at what might help. We had focus group discussions with members of a Patient and Public Involvement Group (PPIG). These are individuals who do not have research expertise, but use their lived experience as patients to influence the research process. This group was formed in 2017, from areas where more people live with social disadvantage. We also interviewed local community leaders. Interviews and focus groups ask open questions, so are a good way to explore what people think about an issue. We found a useful theory about how cultural history affects what people can do. We used this to help us to understand how our findings could improve and widen participation in research within underserved communities.

**What we found:**

We found that trust is very important. There needs to be trust between people and organisations. There are also practical reasons people from underserved communities might not be able to get involved in research. Researchers need to be aware of these things, and work with people from these communities throughout all stages of research. Long term relationships need to develop between institutions and people doing research. Understanding each other’s culture and history makes it easier to work together.

## Background

Research suggests that people from underserved populations, including persons living in poverty and people from ethnic minority communities, have greater risk of poor health, and poorer experiences of using health services [1–3] with the two characteristics combining to create compounded inequity. This was clearly demonstrated during the COVID-19 pandemic, where the impact on minority populations was disproportionate [4]. The majority of primary healthcare research does not have adequate representation from people of these communities [3, 5].

‘Evidence based medicine’ across a range of common health conditions is derived from studies which commonly exclude certain populations within their design, thereby potentially perpetuating health inequalities [6, 7]. The UK National Institute for Health and Care Research (NIHR) produced NIHR-INCLUDE guidance [8] to improve inclusion of underserved groups in research. The importance of building trust with communities already subjected to structural and institutional discrimination is highlighted, but there is a dearth of research about *how* to build trust and mutual understanding to inform inclusive research practise. Many studies describe how they have addressed this issue in their own studies, but fewer have resulted in explicit learning which can be applied by other researchers [9, 10]. One example is a toolkit developed by Farooqi et al. [11] to help researchers maximise ethnic minority recruitment to health research: The final toolkit recommendations were considering the communities which the research needs to involve; effective patient and public involvement; effective recruitment; cultural competence of research procedures; and effective feedback. Recommendations were also made as the result of a systematic review by Bonevski et al. [12], who Identified barriers to involvement in health research for socio-economically disadvantaged groups, and strategies to overcome these. Barriers included mistrust of research within some communities and reliance on technology to collect data. They then developed a set of strategies covering the entire research process from sampling to retention.

A recently published paper by Mitchell et al. 2023 [13] outlines steps to move from tokenism to citizen control in the research process, to increase representation in research:

- 1. Build trust and dialogue by exchange of ideas in a community setting, led by community members.
- 2. Include knowledge sharing about the topic of interest with patients and communities, for example, producing lay summaries of a literature review and bringing in a topic expert for a ‘question and answer’ session.
- 3. Support the development of research skills in communities where it is desired (capacity building).
- 4. Co-create from the outset and at every stage of the research process to include generating and prioritising research questions relevant to the public.

Despite ample research evidence demonstrating that lack of engagement not only resides in under-represented communities but also in research teams and institutions [9], the lack of progress suggests the need for reflective research practice to find solutions to this challenge. We also suggest that the issue needs to be seen in the context of wider societal issues. Local drivers for this work include clearly evidenced health inequalities across the city, and GP practises wishing to be ‘research active’ but finding that studies available to them are not suitably designed for their patient population.

The idea of Cultural Trauma as a mechanism to disrupt health and create disparities is proposed by Subica and Link [10], drawing on Fundamental Cause theory which is a key concept within the Social Determinants of Health literature [10]. Subica and Link suggest that Cultural Trauma, the impact of a dominant oppressive group on the resources of another group, damages three health-protective cultural resources: modes, institutions, and lands, as the underlying cause for health inequalities. *Modes* encompass “*the languages, norms, customs, values, and artefacts*” that create the internal and external worlds of group members. Cultural Trauma disrupts healthy functioning as modes help people to self-regulate when faced with external stressors.

*Institutions* refers to the systems that define social and community life such as family, educational, religious, and health systems. These institutions protect against stress and support health when individuals have a positive status within them. However, when institutions relegate individuals into lower statuses, this creates stress and leads to poorer health. It may be perpetuated through policies which promote inequality and deny access to flexible resources. *Lands* is used to describe material resources such as property, housing, food and transport, that are necessary to maintain health within the particular society within which a person lives. This can be through physical dislocation from native lands via force, genocide, or disease or discriminatory policies that strip groups of material resources.

Several papers addressing under-representation in research have been able to extrapolate experiences with a particular study to the wider research landscape, using theoretical concepts. For example, Rai and colleagues [14] reflect on what it would take to meaningfully attend to ethnicity and race in health research by drawing on experiences in their own study. Their conclusions are strongly informed by a theoretical understanding of issues around race and ethnicity, for example using the phrase, ‘methodological whiteness’.

A paper about reducing social and racial inequalities in obesity by Rosas and Stafford [15], discusses how to engage people from ethnic minority and socio-economically disadvantaged groups in health research, and focusses on the importance of understanding underlying mechanisms, as a building block for addressing the issue. A paper by Asare [16] discusses the concept of social determinants of health (SDH) framework (health and education, the built environment and social and community life) as an explanation for minority patients being less likely to partake in cancer research. This paper proposes a framework which nurses can use to identify where support is needed. Both SDH and Cultural Trauma use Fundamental Cause theory to explain current inequalities, but Cultural Trauma draws together the collective impact of lost resources as a result of oppression. The emphasis in this theory is not on the impact of trauma on their psychological-wellbeing, but on the erosion of health-protective factors within their culture. We suggest that this can be further applied to under-representation of certain groups in health research, which may contribute further to health inequalities.

Working with patients from a Patient and Public Involvement Group (PPIG) based in a low socio-economic area with an ethnically diverse population, and with local community groups that support underserved groups, this study aimed to explore how researchers and research institutions can address inequalities in research, from the perspective of underserved groups. Using Cultural Trauma as a theoretical lens helps to contextualise our findings in the wider social context.

## Methods

We collected data from a focus group with a PPIG, and interviews with community leaders from four voluntary care organisations serving Roma, South Asian, Black, and socio-economically deprived populations. This provides a rich understanding of the barriers and facilitators of participation in research from patient and public perspectives.

### Focus groups and interviews (setting and participants)

A focus group methodology was chosen to collect data from the PPIG group, as focus groups are a well-accepted method in social science and the group process allows participants to identify and clarify their views [17]. Moreover, this was a pre-existing group, who were already comfortable in communicating their views in a group setting. This group was asked to participate as all members live in socio-economically deprived areas, and the group has members from range of ethnic backgrounds and with a range of educational levels.

The decision to do group or individual interviews with community leaders was a pragmatic one, based on the busy schedules of community leaders. Potential participants were purposively sampled via community organisations serving ethnic minority and socio-economically deprived populations.

All participants were contacted via email with a brief description of the research, and asked to contact the researcher (KF) if they would like to take part. They were then sent an information sheet with the opportunity to contact the researcher to ask questions, before returning a signed consent form.

The topic guide for the focus group (see appendix 1) was developed using NIHR-INCLUDE [8] recommendations, and stakeholder consultation.

Focus groups and interviews were facilitated by KF. KF is a female post-doctoral Research Associate, with over 20 years’ experience of qualitative health research, including conducting focus groups and interviews. KF is white British, and a practising Muslim.

In the first focus group, six participants were recruited, with informed consent, from the PPIG. The participants were two men and four women, age between 40 and 75. Three participants were White British, one Black African and two South Asian. All live in socio-economically deprived neighbourhoods.

A focus group lasting 75 min was conducted in a University building, which was easily accessible by both car and public transport. It was audio-recorded, transcribed verbatim, and then identifying information was removed for analysis. KF took field notes to supplement the analysis.

For the interviews, 4 community leaders were recruited from 4 community organisations, to represent a variety of underserved communities (Roma, South Asian, Black, and socio-economically deprived). The aims of the research were discussed with the participants when inviting them. All who were invited, agreed to take part. The participants were 3 women and 1 man, ranging from age 40–70, and their ethnic backgrounds were Roma, South Asian, Black and White British.

Community leaders were presented with and asked to comment on the findings of the rapid analysis from the PPI focus group (see Table 1). Participants were also asked how academics and clinicians may be able to work with community groups to increase participation.

**Table 1:**
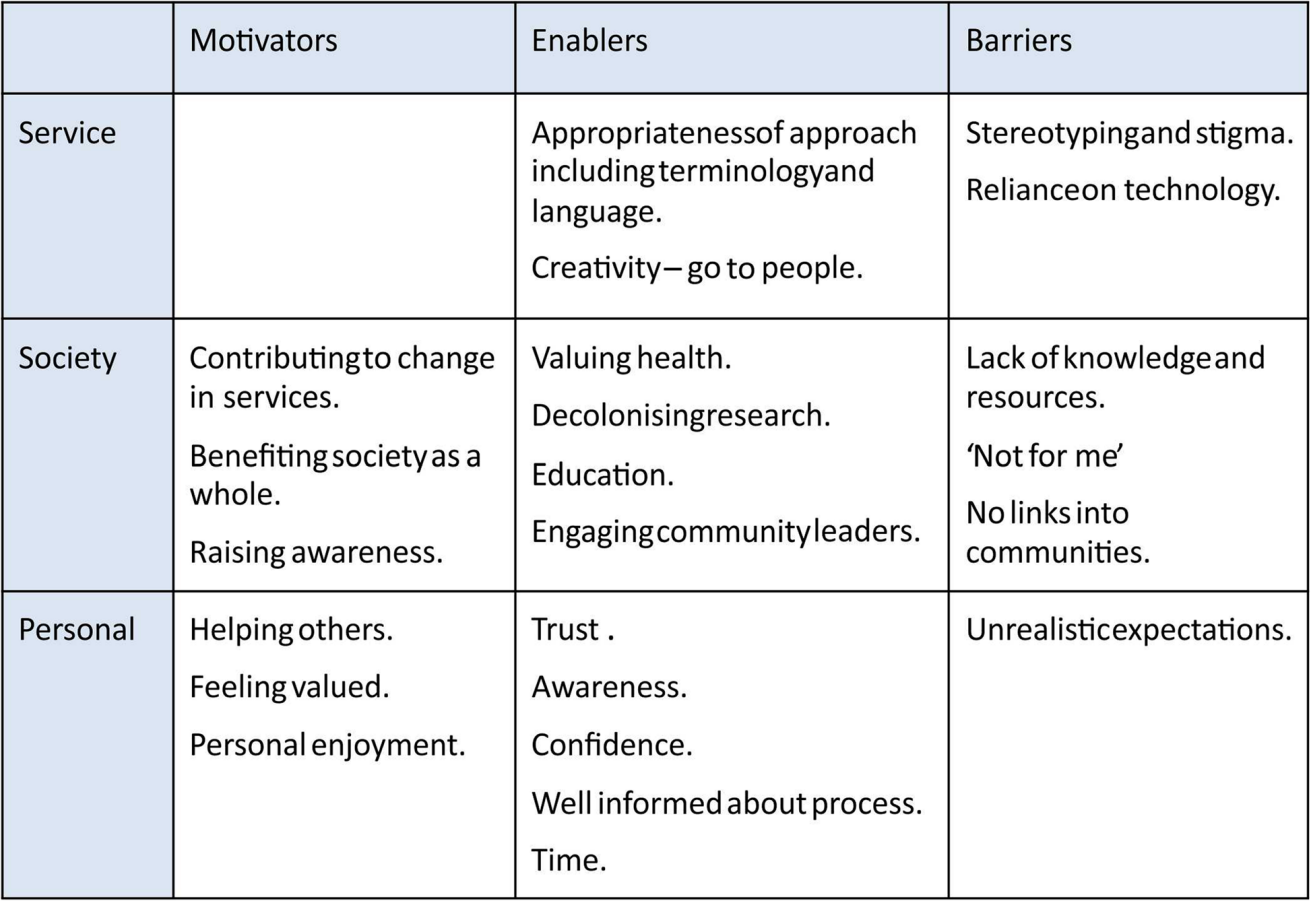
Template from focus group rapid analysis

One group interview (three participants) and an additional single interview took place online, as this was preference of the community leaders. The group interview participants were Black Caribbean, South Asian, and Roma, and provided leadership in organisations serving people from those backgrounds. The individual interviewee was White British, leading a community organisation within a socio-economically deprived area. The researcher (KF) and the participants were the only people in the interviews. The group interview was 69 min long, and the single interview was 40 min long. KF took field notes to supplement the analysis.

KF continually checked her understanding of what was being said in the focus groups and interviews with the participants, which was decided as preferable to sharing the transcripts afterwards, which would have been extremely time consuming for the attendees to read through.

The focus group and interviews did not aim to reach data saturation, but to gather enough data from a variety of perspectives to provide conceptual depth.

### Analysis- template analysis applying cultural trauma

**Fig. 1:**
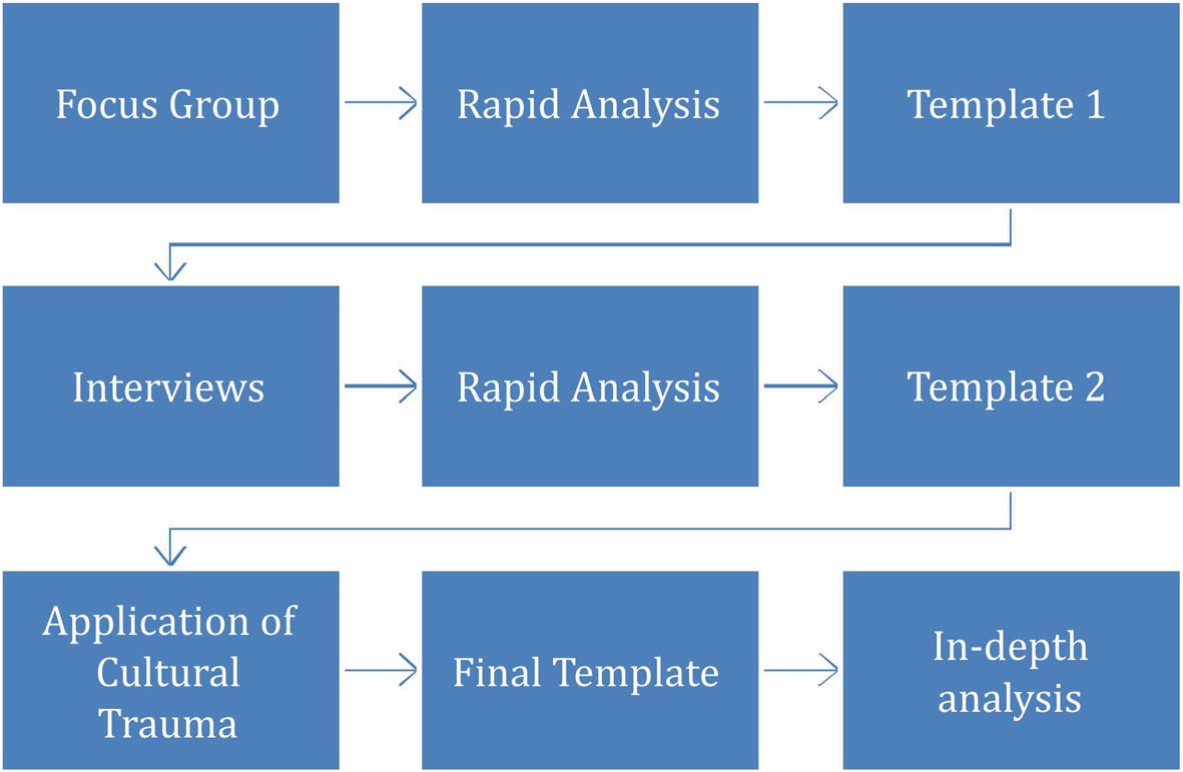
Analysis process

Template analysis involves [18] the development of a coding ‘template’, which summarises themes identified by the researcher(s) as important in a data set, and organises them in a meaningful and useful manner. We used template analysis to allow us to bring in wider social and cultural factors to our understanding of our findings, and we achieved this by applying the Cultural Trauma concept. Figure 1 illustrates how we created the template.

The template for analysis was created by rapidly analysing the focus group and interview transcripts to create initial templates (see Tables 1 and 2).

**Table 2:**
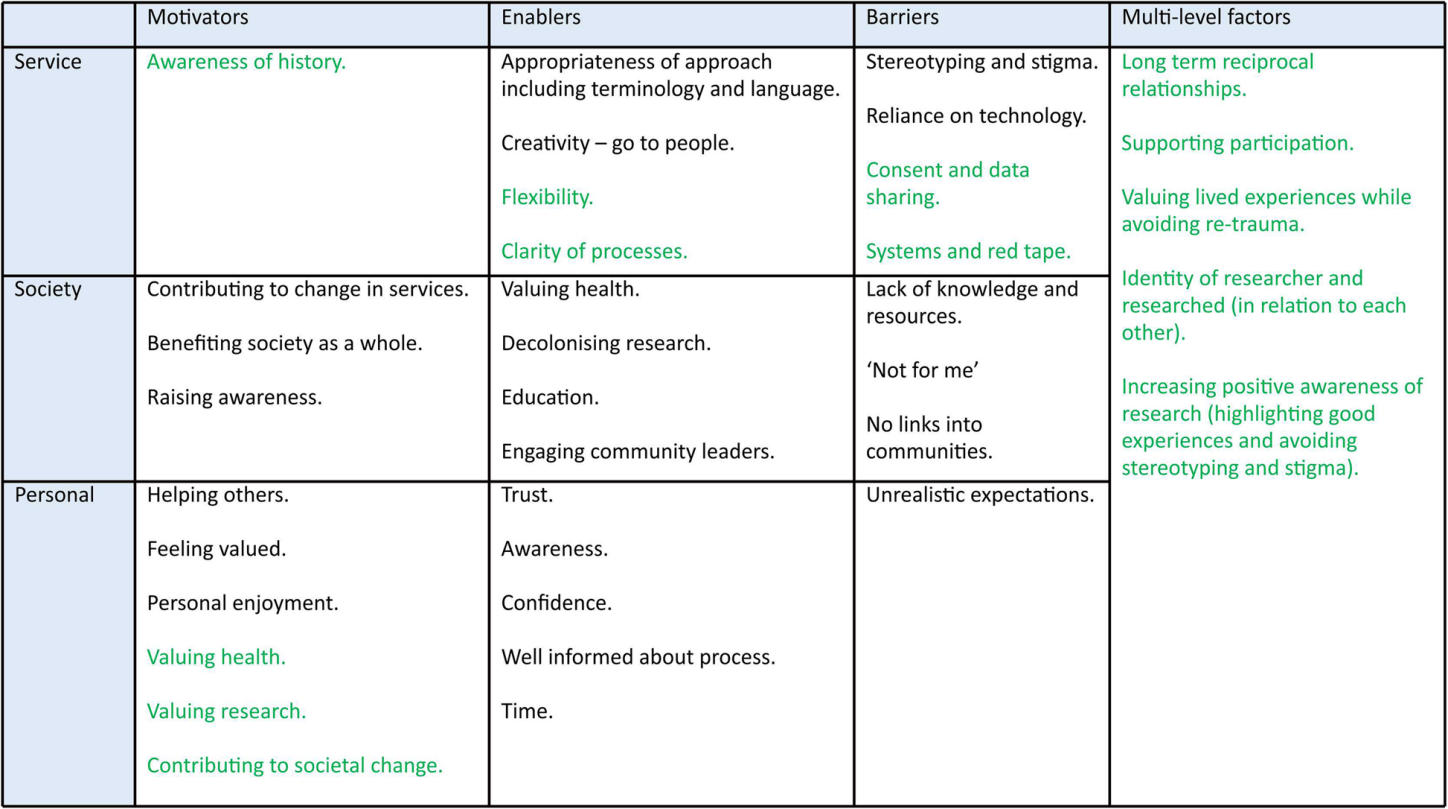
Template from focus group and interviews rapid analysis

This was carried out by three members of the research team (KF, EL, JW). The themes were then applied to the three elements of Cultural Trauma (modes, institutions, lands), which helped to give a wider context to the findings (by KF, IH, CM). It was important to develop the initial templates from the study findings, to ensure data was not forced into the theoretical concept.

A detailed analysis of the focus group and interviews was then carried out according to this final model (by IH, KF), using NVivo software. The final model was presented to the PPIG, who confirmed their views were adequately represented and the model made sense to them.

**Fig. 2:**
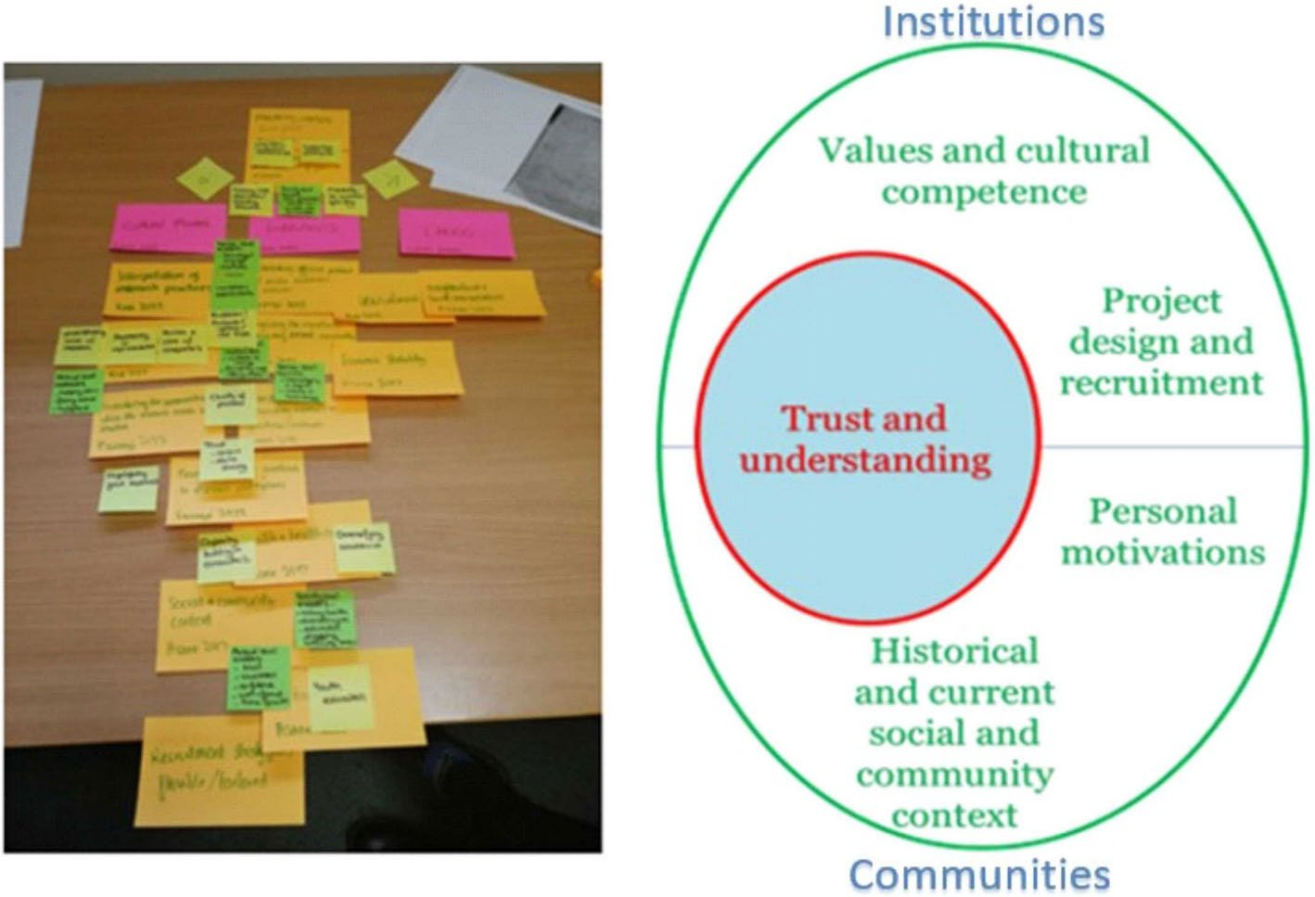
Applying Cultural Trauma to the template

**Fig. 3:**
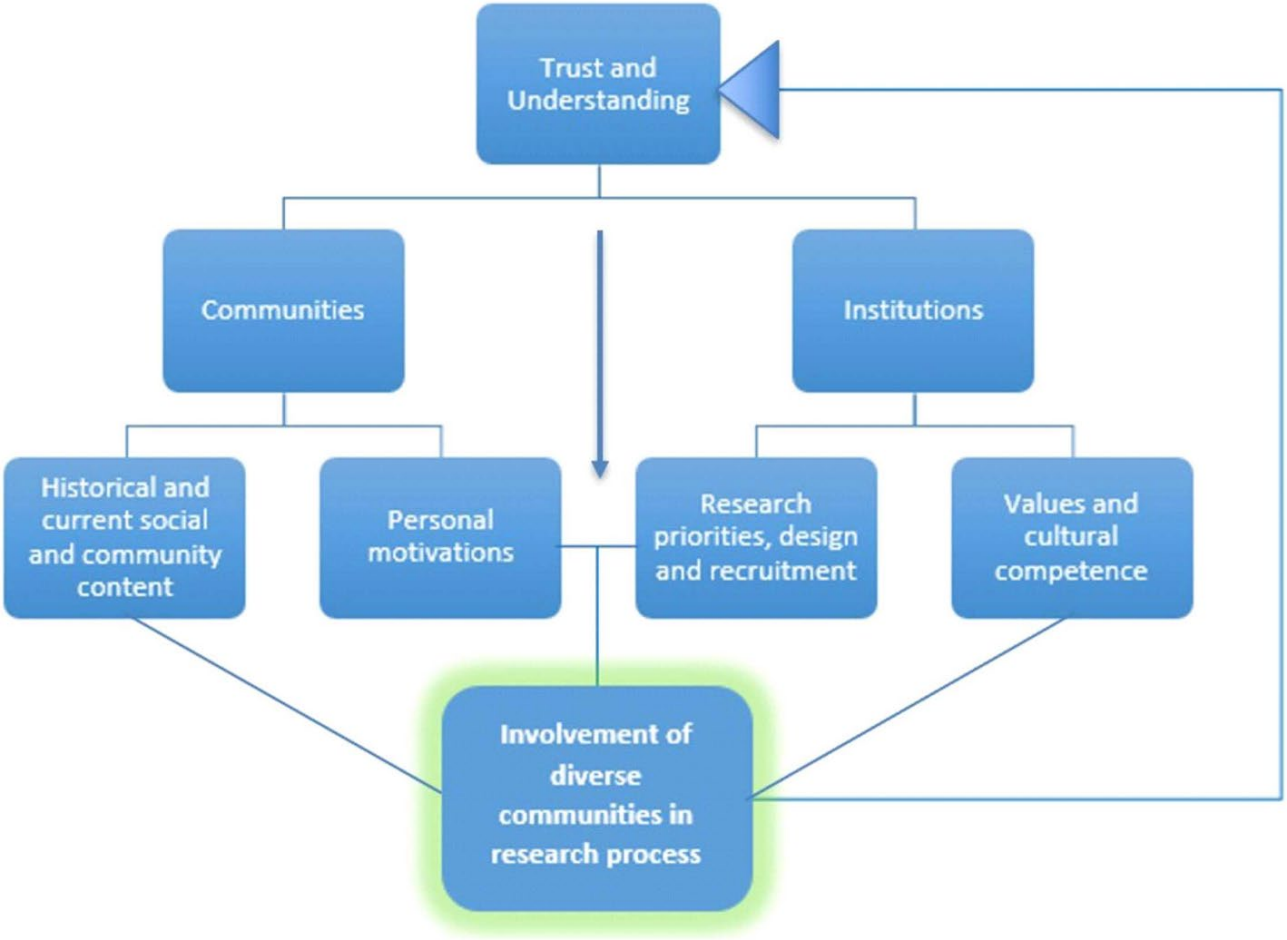
The process of research involvement in underserved communities

Figure 2 shows how the themes were developed. The three elements of Cultural Trauma [10] are shown below in the photo on the pink Post-It notes, and key concepts from the background literature are shown on orange Post-It notes. We then took the themes from our rapid analysis (shown on the small square Post-It notes) and arranged them with or between the three elements of Cultural Trauma. This process started to form a picture. There was a cluster of themes between cultural modes and institutions, indicating that the interaction between these two elements is important. Other themes formed a circle around them, indicating factors that were influential in creating this interaction, bringing us to the final model. Where these are presented in the egg-shaped diagram in Fig. 3, the top half of the egg contains factors relating to institutions, and the bottom half shows factors related to communities.

An in-depth analysis based on this template deepened our understanding of how themes related to each other, and this is represented in Fig. 3.

## Results

### Trust and understanding

Our findings from the interviews were clustered around the Cultural Trauma elements of *modes* and *institutions*, showing tensions between these, and the importance of trust and understanding. An exploration of themes clustered between ‘Modes’ and ‘Institutions’ suggested that trust between under-served communities and institutions (in this case health and academic institutions) was of utmost importance regarding research participation and was often absent.

Research happens within a wider social context. This context will influence the research process, and the research process cannot be understood without reference to the wider social context. Historical and societal issues will influence communities and therefore how individuals act. Institutions and their relationship with communities will be influenced by these same issues, and will influence how researchers act.

Trust and understanding is therefore for an over-arching theme, which relates to each of the subthemes: ‘Values and Cultural Competence’ and ‘Research priorities, design and recruitment’ which allow us to explore this issue from the point of view of the researcher and their institutions; and, ‘Historical and current social & community context’ and ‘Personal motivations’ in which we understand this from the point of view of under-served communities.

### Values and cultural competence

Our findings suggest that values and cultural competence are crucial to building trust and understanding between academic institutions and under-served communities. This was both true at a larger societal scale, and a more local and project specific level. At a larger scale for example, the need for decolonising of the curriculum was mentioned, reflecting the feeling that contributions from non-white academics, and other ways of knowing or cultural interpretations of health had been removed from our understanding of many subjects taught today:

*“All the contributions from those previous civilisations, they have contributed to what we know as knowledge today…So people like, different people, so everyone can see that their ancestors did something. It gives them confidence that we too, we can” (PPI contributor - PPIC)*

This is an example of the ‘institutions’ aspect of cultural trauma, where policies and denied access have impacted upon the current perceptions of under-served communities within academic institutions.

An observation was made in both focus groups and interviews that research teams are predominantly white and middle class. Where those being researched are from socio-economically deprived or ethnic minority backgrounds, this may reinforce established power dynamics. Diversifying research teams was seen as desirable, but it was acknowledged that people from underserved communities may be less likely to establish academic careers, creating a circular problem:

*“if a community member doesn’t feel that they can go to university, or that that’s even an option for them, the last thing is… I don’t see that link between them contributing to research” (Community Leader- CL)*

This relates to both *modes* and *institutions* within Cultural Trauma – historically, access to educational institutions has been explicitly denied to under-represented groups, and is currently denied implicitly. The process of this has also devalued the *modes* of under-served groups (e.g. languages and artefacts). Engaging with community leaders for research recruitment was suggested as a solution to overcome this issue. In the light of Cultural Trauma, the mechanism behind this may be to increase positive regard of the institution towards the under-served group, and facilitate access to the under-served group.

However, the community leaders themselves recognised the limitations of this approach from their perspective, and the difficulties this can cause for community organisations:

*“it is very difficult for us to keep being asked to participate […] it’s just like, oh, can you get us so and so, can you get us so and so, can you get so and so? We’ll give you a little budget for it, and that’s it. And then we go out. And it costs a lot more of our time” (CL)*

It was recognised that ‘helicoptering in’ could be very damaging. This issue relates to *lands* and can be seen as a result of indirectly discriminating policies impacting upon the distribution of flexible resources. Community leaders unanimously wanted ongoing relationships between community organisations and Universities, which were not only transactional:

“*To make it a good process, there should be some kind of good partnership going”(CL)*

In the longer term, capacity building in communities was suggested as a way of breaking the cycle and ensuring that people understand the role and value of health research:

*“And that’s the education, it’s the awareness, it’s as much getting that out there to start that little pea on the roll until it becomes bigger and bigger and bigger and the information, but there’s so much information and education about services that needs to still happen”* (PPIC)

This would impact upon the *lands* and *institutions* elements of Cultural Trauma, by redirecting resources and building positive regard within academic and health institutions, for the under-served group.

The values of the researcher and research team, and their cultural competence, are essential in enabling inclusive research. The first step in this process is to have researchers who genuinely see that engaging collaboratively with communities can enhance the research process:

“So it’s just this kind of how can we see people as humans that have the capacity to find the solutions and offer solutions for a lot of the things that are the problems? They might not have the confidence, they might not have the resources or the skills really to… But they know what they need and they just need a little bit of scaffolding and support to get there” (CL)

Where this is successfully achieved, it may impact upon *modes*, by valuing and highlighting the knowledge and skills present within under-served communities, and increase positive regard within institutions. This involves an awareness of the historical and cultural issues relating to specific communities. This awareness is necessary for understanding how certain elements of the research process may be triggering for particular groups, relating to the *modes* element of Cultural Trauma, which acknowledges that self-regulation against stress can be damaged:

“What might look very innocent for us, and it is about respecting them, might trigger some trauma for them” (CL)

This awareness was seen as something that would result from extended engagement with communities, and would not be achieved in a one-off training session:

“If we think of these things as an event as opposed to a process, we’ve got it wrong. It’s not going to be… It’s taken years to get to the point where we are, so we’re not going to turn the ship around overnight. So I think it is about continuing to make those networks, to make those connections, to make real the community partnerships that universities are attempting to do now. But not to take your eye off the ball and think, well, we’ve done that now.”(CL).

This statement has implications for institutions, in how they may control or impact resources available to communities, speaking to the ‘lands’ element of CT.

### Historical and current social & community context

The significance of historical cultural factors and their impact on trust and understanding was striking during the focus group with the PPIG. These are issues of race and class in general, which may have contributed to issues of stigma and ‘otherness’ that have embedded a culture in which willingness to participate in research is unlikely. Historical exclusion from and exploitation in health research caused disengagement and therefore a present lack of awareness. This is clearly embedded in the *modes* aspect of Cultural Trauma, and directly impacts upon how people relate to academic and health institutions currently. In addition, community leaders spoke about specific scandals around research, such as the Tuskagee study in America involving Black men, as being very much in the forefronts of people’s minds when they were asked to participate in health research:

*“they’ve not been reassured that the current ethics and current standards make that kind of thing less likely to happen” (CL)*

Not only do policies need to change to protect under-served groups, but this needs to be demonstrated and communicated. More general health injustices also had an ongoing influence over people’s response to research, even if they occurred in other countries:

*“If we’re talking about in Slovakia, they still sterilise women, so of course there’s going to be a mistrust of doctors here in the UK. It’s not that far removed for them” (CL)*

Practical issues such as access to technology, and literacy levels, also impact upon individual capacity to take part, fitting with the *lands* element of Cultural Trauma. However, more time was spent discussing more abstract concepts, such as valuing health and awareness of research. which was felt to be lacking or very basic:

*“yes, there is an awareness of research but I think only what they see on the television and that’s people with the pipettes and putting things in test tubes” (PPIC)*

Not only do communities need an awareness of research, but more specifically, how it may affect, and therefore relate to them:

*“I think most people, I mean, if you talk to them about research, they probably think that research is not something that affects them. Like you say, it’s like doctors and, you know, people who are educated, that’s something they’ll do, so it’s nothing got to do with them. So they won’t really be interested or want to join in” (PPIC).*

This statement demonstrates the impact of denied access over time.

### Personal motivations

The factors described above will influence people’s responses to research invitations. During the focus group with the PPIG, we explored what factors had motivated them to become involved in research. Most stated that they valued the opportunity to improve services, and understood that the patient perspective was necessary in addition to the expertise of the researchers:

*“That expertise, we public and members of the public, our common knowledge enhances their expertise. And if that common knowledge is not there from our perspective, that expertise is limited.” (PPIC)*

This suggests that the people in the PPIG group had overcome CT to some degree, in order to realise the value of their perspective, and how it may have a positive impact. Contributing in this way gave them a feeling of doing something to improve society in general:

*“I just thought if I could make one tiny, tiny, tiny bit of difference, then, you know, I’d feel better” (PPIC)*

It may also be, in itself, a way of addressing Cultural Trauma. The statement above suggests that the PPIC is aware of the size of the problem faced by under-served communities in regard to health inequality. By contributing, they may be attempting to repair damage to the *mode* element, by learning to value their own contribution.

### Research priorities, design and recruitment

The values and cultural competence of the research team was seen as manifesting in the research process. The PPIG, who are experienced in engaging with researchers, talked about knowing when they are part of a ‘tick box exercise’ as opposed to when engagement is genuine. A ‘tick box exercise’, where engagement is superficial and has no real influence on the research process, is an example of how *institutions* may continue to deny access, despite updated policies which aim to enhance participation.

There was a tension between valuing lived experience, and the risk of re-traumatising people through the discussion of difficult life events:

“Increasingly, I’m not approaching the people we work with, to say, share your lived experience, because it’s just I’ve seen it re-traumatise.” (CL)

Here we see the cumulative effect of Cultural Trauma, where past trauma and its impact on self-regulation against stress, prevents active participation in the here and now.

Awareness of the language needs of the people that researchers want to involve in research is essential, including literacy levels and different forms of communication. It was suggested that researchers need to be more creative in the way they undertake the information and consent process around research, relying less on technology, and more on traditional forms of communication, approaching communities in their own areas and environments:

“the people that you want really are not going to sit checking their emails. It’s more conversations, the traditional forms of communication that we need to fall back on.” (PPIC)

This both suggests learning to value the *modes* of particular under-served groups, and also *their institutions*.

A key part of moving away from the ‘helicoptering in’ approach, was to ensure that people participating in research had feedback regarding the study findings, and impact of their input on the research process:

“I don’t think people would mind if they didn’t go ahead or there were some problems. But as long as they got feedback so they feel appreciated, they feel that next time they want to do more to help, be involved”. (PPIC).

The fact that this is not seen as common practise is yet more evidence of denied access to *institutions.* Long term relationships with communities were seen as the ideal model, in which this would happen naturally:

“if you’re doing this kind of research, you’re going to have to come back to the community and it should be an iterative process. And that isn’t always the model. And I understand it’s resources and time and time is currency. However, I do feel that when you invest, and if you’re really committed into finding and gathering quality data, then you need to invest that time in trying to get an understanding of the community.” (CL).

This CL directly refers to the flexible resources alluded to in *lands. ‘Resources and time and time is currency’* acknowledges that academic and health institutions often choose *not* to invest limited resources in under-served communities. There was a more general message coming through, that the necessary actions would come from a mind-set which genuinely valued and respected people’s experience, the factors encompassed by the *modes* aspect of Cultural Trauma:

“no matter who you come across in life, even the guys that live on the street, when you interact with that person, you enrich one another consciously and unconsciously. You learn from that person, that person learns from you. Whether you accept it or not, consciously and unconsciously.” (PPIC).

Reimbursement of community member’s time was problematic. Community leaders and PPI members noted issues with delays in reimbursement, and the limitations of offering vouchers, which have negative connotations within some communities, such as being associated with being in need, or on a low income. This falls within the *lands* elements of Cultural Trauma, but also overlaps with *modes* in that there are implications for particular communities of means of payments, in relation to their values and customs. These issues cause inconvenience and undermine the relationship between researchers and communities:

*“no-one should have to wait six to eight weeks to be paid for something. And that’s what we always say, well, because of the paperwork or… But that’s not fair, we wouldn’t wait six to eight weeks to be paid on a salary, so why should we allow freelancers or community connectors or organisations, expect them to do that?” (CL)*

While paying community organisations for their input was recognised as important, the amount given was often insufficient to cover the actual time and resources of the community group in engaging with the research, putting further stress on the flexible resources covered in the *lands* element of Cultural Trauma. Community leaders made it clear that any genuine engagement means involvement from the earliest stages, and in on ongoing relationship:

*“We’d like to be part of shaping and implementing as well and be part of… To make it a good process, there should be some kind of good partnership going on with the university and communities” (CL)*

## Discussion

### Summary of findings

The ability for research to be inclusive depends upon trust between academic and health institutions and the communities that research hopes to engage. The impact of Cultural Trauma was evident in the four subthemes, demonstrating the wider context in which this trust needs to be established. The concept of Cultural Trauma aids us in understanding how the lack of representation of under-served communities in research relates to wider aspects of these community’s experiences. We are able to see how damage to *modes, institutions and lands* directly impacts upon the current situation, from the point of view of researchers and their institutions, and under-served communities.

The barriers to involvement in research by people from underserved communities that we found in our focus groups support those present in other studies, particularly in Bonevski et al.’s systematic review [12]. Further, we found that the socio-cultural context was extremely influential in how particular groups may respond to research, and that it is important that researchers be aware of this. Similarly, Rosas and Stafford [15] emphasised the importance of understanding underlying mechanisms, and Farooqi et al. [11] emphasised the importance of cultural competence of research teams, in addition to the more practical strategies needed. Our findings also suggested that the predominantly ‘white’ nature of research teams was itself a barrier to inclusive research, a problem described by Rai et al. [14] as ‘methodological whiteness’.

Taken as a whole, our findings emphasise the importance of seeing the research process within a wider context, and we found Cultural Trauma to be a useful framework for understanding the issue. The Cultural Trauma concept suggests damage to three health-protective cultural resources: *modes, institutions,* and *lands*, as resulting in Cultural Trauma, an underlying cause of health inequalities [10]. In this paper, we have demonstrated how this can be further applied to under-representation of certain groups in health research, by using the model as a lens by which to understand our data. This enables us to see the circular impact created by under-representation of underserved groups in research, which further exacerbates health inequalities. The concept of Cultural Trauma helps to frame the under-representation of certain groups in research in relation to broader societal issues, without which it cannot be properly understood.

### Strengths and limitations

In the focus groups and interviews we engaged directly with people from socio-economically deprived areas, and from a variety of ethnic backgrounds. We also engaged with community leaders who had a wider view of the relevant issues.

While all of the participants involved in the focus group were from socio-economically deprived backgrounds, half were White British, and therefore could be seen as forming part of the ‘dominant group’ by other people in the group that were from ethnic minority backgrounds. This could have influenced what people felt able to say in the group. However, the effect may have been somewhat mitigated by it being a pre-established group, who had many conversations about class, race and inequality, and therefore are likely to have been more comfortable expressing their views with each-other.

This study was undertaken in a single urban setting, but with people from socio-economically deprived and ethnic minority backgrounds which makes a contribution to a growing body of literature in this area. However, we need to acknowledge that those who participated are likely to be among the most literate and health aware within their communities.

The lead researcher (KF) who facilitated the focus groups, is well known to the PPIG (and present at most of their meetings), and this may have influenced the responses they felt able to give. However, the group has an established ethos of challenging the idea but not the person, and the group appeared comfortable with disagreeing with her. This relationship may also have influenced the data as some things may have been implicitly assumed and so not verbalised.

## Conclusions

Lack of representation in primary healthcare research is part of wider societal inequity but there are actions that researchers can take to improve inclusivity in primary healthcare research, thereby improving the quality of data and making evidence-based practise more accurate and valuable within all communities. This could contribute significantly to the reduction of health inequalities.

Whilst the NIHR-INCLUDE Roadmap [8] provides a good structure for an inclusive research process, prolonged engagement between academic institutions and communities over time and across multiple research projects, and individual researcher reflexivity with respect to differential power and cultural competencies are also crucial to widening participation in research.

Study data collection is the end point of a long process of prioritisation, design and funding procurement, and change needs to occur at all points in that process for real change to happen. If the organisations which currently ‘own’ research, wish research to be representative of the UK population, those organisations need to be willing to disrupt their philosophies and processes.

## Recommendations

Research institutions and those who represent them should:

- Take responsibility for lack of diverse representation in primary healthcare research.
- Build trust and understanding with communities by:
  o Developing cultural competence
  o Diversifying research teams
  o Engaging with community leaders
- Invest time and money in long-term reciprocal relationships.
- Recognize untapped potential within communities and build capacity for engaging in research.
- Ensure that research is not ‘exclusive by design’, for example, considerations around language, digital exclusion of ways of recruiting.

It is also essential to embed PPI which represents and includes those from underserved communities throughout the research cycle, and strategies need to be in place to ensure this happens in a genuine way rather than a ‘tick-box exercise’. Utilising participatory methodologies demonstrates a commitment to inclusive study design from the outset.

## Data Availability

No datasets were generated or analysed during the current study.

## Appendix

Focus group topic guide PPI group

1. Why do you think health research is important?

2. Do you think that most people understand the importance of health research?

3. What motivated you to get involved?

4. What my motivate others in your area/community?

5. What are the barriers to people getting involved in health research?

Prompts: different ethnic backgrounds, levels of education, struggling financially.

1. What things can researchers do, to encourage/make it easier for people to get involved?

## Abbreviations

PPI: Patient and Public Involvement
UK: United Kingdom
PPIG: Patient and Public Involvement Group
NIHR: National Institute for Health Research
PPIC: Patient and Public Contributor
CL: Community Leader
SDH: Social determinants of health

## References

[1] Marmot MAJ, Boyce T, Goldblatt P, Morrison J (2020). Health Equity in England_The Marmot Review 10 Years On In. BMJ.

[2] Marmot M AJ, Boyce T, Goldblatt P, Herd E, Morrison J. <Build-back-fairer-the-COVID-19-Marmot-review. 2020. https://www.instituteofhealthequity.org/resources-reports/build-back-fairer-the-covid-19-marmot-review/build-back-fairer-the-covid-19-marmot-review-full-report.pdf. Accessed 02 Aug 2024.

[3] Ralaigh V. The health of people from ethnic minority groups in England [Long Read]. The Kings Fund. 2023. https://www.kingsfund.org.uk/publications/health-people-ethnic-minority-groups-england. Accessed 02 July 2024.

[4] Ma KC, Menkir TF, Kissler S, Grad YH, Lipsitch M (2021). Modeling the impaCt of racial and ethnic disparities on COVID-19 epidemic dynamics. Elife.

[5] Bodicoat DH, Routen AC, Willis A (2021). Promoting inclusion in clinical trials—a rapid review of the literature and recommendations for aCtion. Trials.

[6] Khunti K, Gray LJ, Skinner T, Carey ME, Realf K, Dallosso H et al. EffeCtiveness of a diabetes education and self management programme (DESMOND) for people with newly diagnosed type 2 diabetes mellitus: three year follow-up of a cluster randomised controlled trial in primary care BMJ 344 :e2333 2021. 10.1136/bmj.e2333. 10.1136/bmj.e2333 PMC3339877 22539172

[7] McManus RJ, Little P, Stuart B, Morton K, Raftery J, Kelly J et al. Home and Online Management and Evaluation of Blood Pressure (HOME BP) using a digital intervention in poorly controlled hypertension: randomised controlled trial BMJ; 372 :m4858. 2021 10.1136/bmj.m4858 10.1136/bmj.m4858 PMC7814507 33468518

[8] NIHR. Improving inclusion of under-served groups in clinical research: Guidance from INCLUDE project Online. In: NIHR. 2020. https://www.nihr.ac.uk/documents/improving-inclusion-of-under-served-groups-in-clinical-research-guidance-from-include-projeCt/25435.

[9] Armstrong K, Ritchie C (2022). Research Participation in Marginalized Communities — Overcoming Barriers. N Engl J Med.

[10] Subica AM, Link BG (2022). Cultural trauma as a fundamental cause of health disparities. Soc Sci Med.

[11] Farooqi A, Jutlla K, Raghavan R (2022). Developing a toolkit for increasing the participation of Black, Asian and minority ethnic communities in health and social care research. BMC Med Res Methodol.

[12] Bonevski B, Randell M, Paul C, Chapman K, Twyman L, Bryant J (2014). Reaching the hard-to-reach: a systematic review of strategies for improving health and medical research with socially disadvantaged groups. BMC Med Res Methodol.

[13] Mitchell C, Fryer K, Guess N, Aminu H, Jackson B, Gordon A, Reynolds J, Huang Q, Jayasooriya S, Mawson R, Lawy T, Linton E, Brown J (2023). Underserved “Deep End” populations: a critical analysis addressing the power imbalance in research. The British Journal of General PraCtice : The Journal of the Royal College of General PraCtitioners.

[14] Rai T, Hinton L, McManus RJ, Pope C (2022). What would it take to meaningfully attend to ethnicity and race in health research? Learning from a trial intervention development study. Sociol Health Illn.

[15] Rosas L, Stafford R (2012). Practical research strategies for reducing social and racial/ethnic disparities in obesity.. Int J Obes Suppl.

[16] Asare M, Flannery M, Kamen C, editors. Social determinants of health: a framework for studying cancer health disparities and minority participation in research. Oncol Nurs Forum. 2;44(1):20–23. 2017. 10.1188/17.ONF.20-23. 10.1188/17.ONF.20-23 PMC5583708 28060469

[17] Onwuegbuzie AJ, Dickinson WB, Leech NL, Zoran AG. A Qualitative Framework for Collecting and Analyzing Data in Focus Group Research. Int J Qual Methods. 2009;8(3):1-21. 10.1177/160940690900800301.

[18] Brooks J, McCluskey S, Turley E, King N. The Utility of Template Analysis in Qualitative Psychology Research. Qual Res Psychol. 2015;12(2):202–22. 10.1080/14780887.2014.955224 PMC4960514 27499705

